# Cystoid Macular Edema: A Rare Adverse Reaction to Rituximab

**DOI:** 10.7759/cureus.52867

**Published:** 2024-01-24

**Authors:** Ana Isabel Machado, Mariana Marques, Marina Vieira

**Affiliations:** 1 Internal Medicine, Hospital de Braga, Braga, PRT; 2 Internal Medicine, Hospital Vila Franca de Xira, Vila Franca de Xira, PRT; 3 Nephrology, Hospital de Braga, Braga, PRT

**Keywords:** nephrotic syndrome, adverse drug events, membranous glomerulonephritis, cystoid macular edema, rituximab

## Abstract

Membranous glomerulonephritis is the leading cause of nephrotic syndrome in non-diabetic Caucasian adults. For patients at risk of progressing to end-stage renal disease, immunosuppression, particularly rituximab, is the recommended treatment. While extremely rare, cases of cystoid macular edema associated with rituximab have been documented in the literature.

In this report, we present the case of a 54-year-old male with membranous glomerulonephritis at a high risk of progressing to end-stage renal disease who experienced cystoid macular edema hours after receiving rituximab infusion. Following the discontinuation of the medication, the patient spontaneously recovered visual acuity without the need for any targeted therapy.

## Introduction

Membranous glomerulonephritis (MGN) is a kidney disorder characterized by inflammation and damage to the glomerular membranes within the kidneys and is one of the leading causes of nephrotic syndrome in adults [1]. MGN is primarily an autoimmune disease where the body's immune system mistakenly attacks the glomerular basement membrane, leading to thickening and disruption of its function [1].

Patients with MGN often present with proteinuria, edema, and hypoalbuminemia. Diagnosis typically involves a kidney biopsy, which reveals characteristic thickening of the glomerular basement membrane under a microscope [2]. In approximately 80% of patients, the condition is attributed to circulating autoantibodies against podocyte antigens, leading to what is known as primary MNG. The rest of the cases are often associated with underlying conditions such as systemic lupus erythematosus, infections, or exposure to certain medications or toxins [1].

The management of MGN focuses primarily on addressing the underlying cause and controlling symptoms [3]. Angiotensin-converting enzyme (ACE) inhibitors may be used to reduce proteinuria and protect kidney function. While the majority of patients achieve spontaneous remission within six months, some patients progress to end-stage renal disease (ESRD), requiring the use of immunosuppressive drugs in selected patients [1,3].

Rituximab (RTX) is a monoclonal antibody that targets and depletes B-cells in the immune system, which play a role in the inflammation associated with MGN [4]. Therefore it’s one of the most widely accepted therapeutic options for managing this disease, despite its adverse effects [3,5].

Cystoid macular edema (CME) is a condition that affects the macula, the central part of the retina responsible for sharp, central vision. It is characterized by the accumulation of fluid within the layers of the macula, leading to swelling and distortion of vision [6].

CME can have various underlying causes, including eye surgery (such as cataract surgery), eye inflammation (uveitis), diabetic retinopathy, age-related macular degeneration, and other retinal diseases [6]. Diagnosis of CME is typically made through a comprehensive eye examination, which may include optical coherence tomography (OCT) to visualize macular changes [6].

Treatment options for CME depend on the underlying cause. Management may involve addressing the root cause, using anti-inflammatory medications, or administering intraocular injections. In some cases, laser therapy or surgical intervention may be necessary to reduce fluid accumulation and improve vision [6].

We present a case of bilateral CME following RTX infusion. This case report holds considerable significance as it illuminates the exceptionally rare incidence of cystoid macular edema as an adverse effect of RTX, a phenomenon documented in just five cases worldwide [7-10]. Furthermore, it underscores the exceptional nature of our case, where full restoration of visual acuity was attained without targeted therapy, thereby providing invaluable insights for the comprehension and management of this uncommon complication.

## Case presentation

A 54-year-old independent male was referred to the nephrology outpatient clinic due to nephrotic syndrome of approximately one-year duration, with progressive worsening dyspnea, orthopnea, fatigue, peripheral edema, and foamy urine. The patient had a medical history of hypertension, dyslipidemia, and ischemic heart disease. He had been taking lisinopril at a dosage of 20 mg/day for approximately seven months, as initiated by the attending physician, which was the maximum tolerated dose due to episodes of hypotension.

The laboratory findings revealed altered renal function, with decreased serum albumin concentration, significant proteinuria, and albuminuria. Anti-phospholipase A2 receptor antibody (PLA2Rab) was positive, without any other notable abnormalities (Table 1). 

**Table 1:** Laboratory workup at the first nephrology consultation and four months after rituximab

	First consultation	Four months after RTX	Normal Range
Creatinine (mg/dl)	1.3	1.3	0.60-1.10
Urea (mg/dl)	140	67	19-49
Albumin (g/dL)	2.8	4.2	3.4-5.0
Proteinuria (g/24h)	17.09	1.09	0.05-0.08
Albuminuria (mg/24h)	8845	600	<30
PLA2Rab (U/mL)	88.2	--	<20

**Figure 1:**
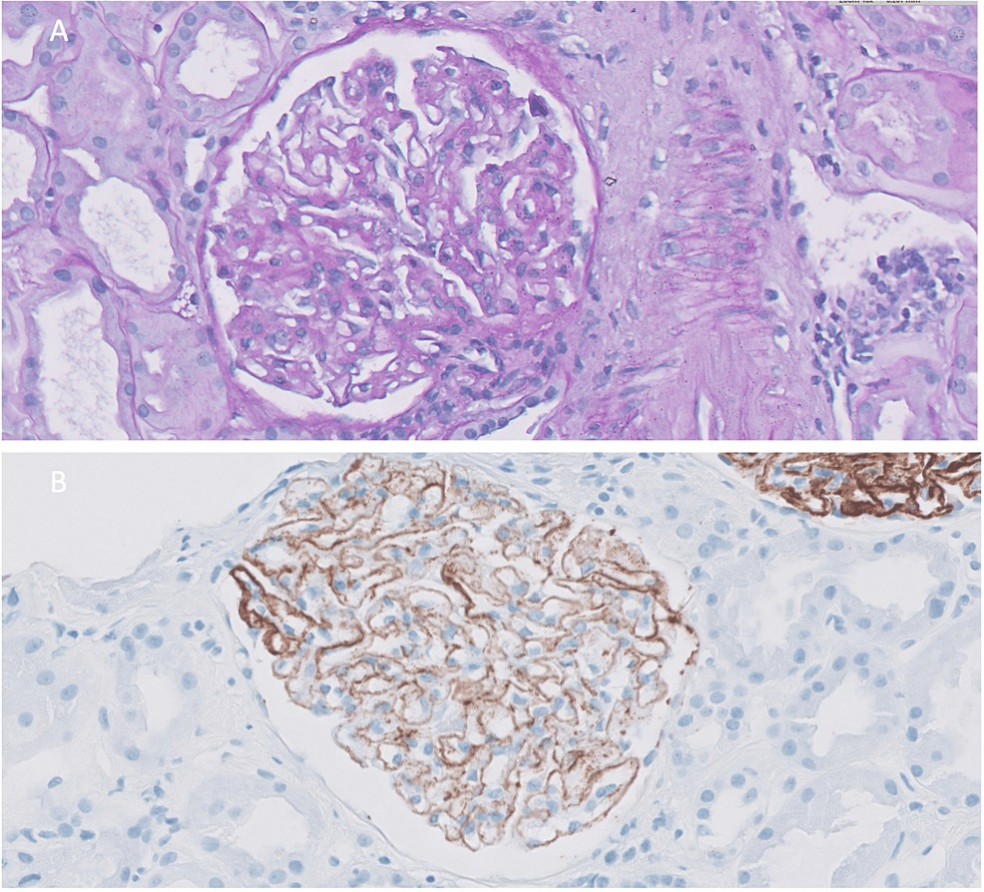
Membranous glomerulonephrits. Glomerulus with thickened basement membranes (1A - PAS stain 400x). Membranous and granular staining for IgG4 (1B- IgG4 400x).

The diagnosis of primary MGN was supported by renal biopsy. Glomeruli had thickened glomerular basement membranes; immunofluorescence showed membranous and granular immunoglobulin G (IgG) deposits with IgG4 expression (Figure 1). 

Further comprehensive assessments, including prior medication history, infectious serologies, autoimmune studies, chest X-ray, renal ultrasound, and malignancy screening, were negative for other causes of MGN. Immunotherapy with oral prednisolone (1 mg/kg/day) and intravenous RTX (375 mg/m2, one dose weekly for one month) was initiated.

**Figure 2:**
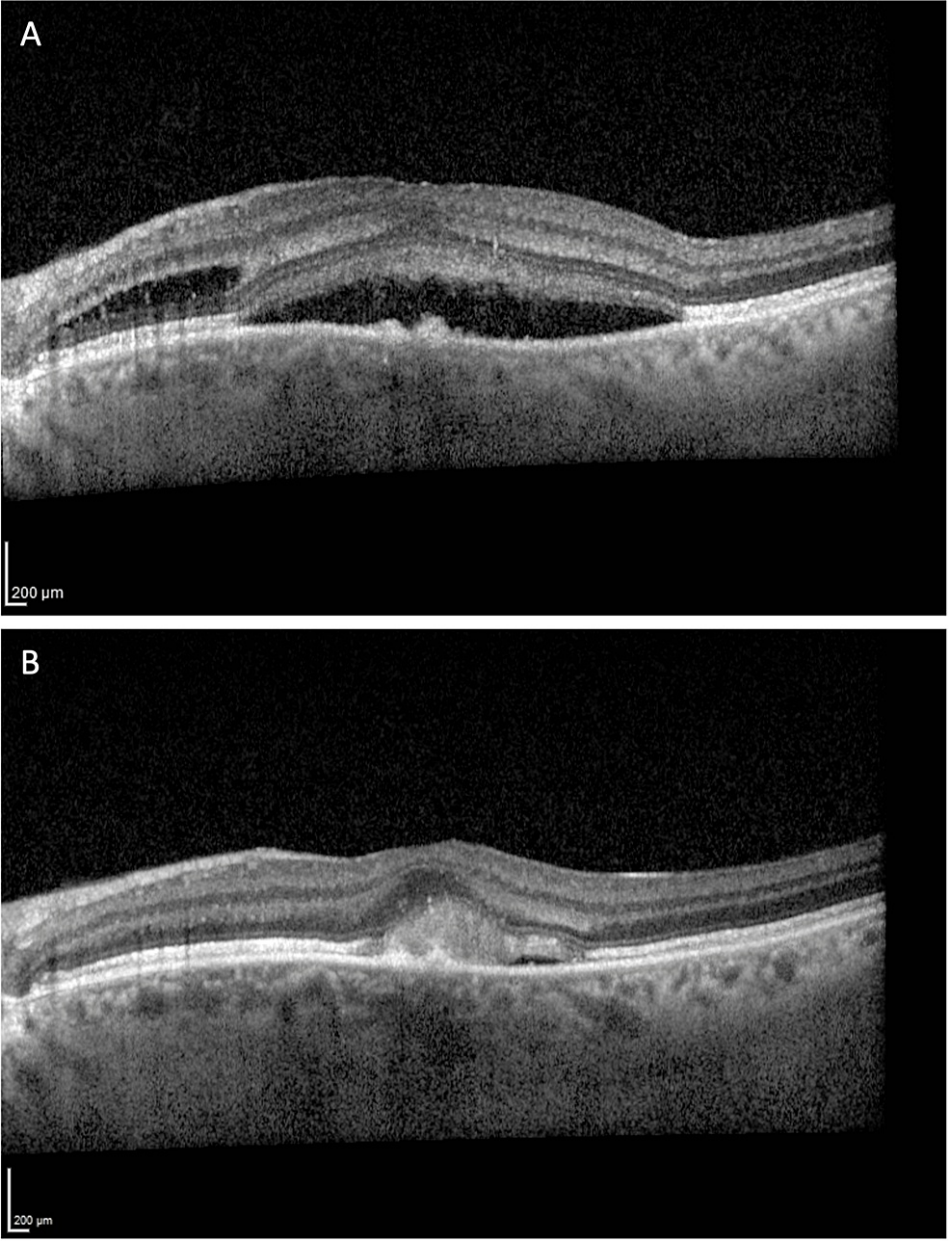
Optical coherence tomography scans of the left eye at the initial presentation (A) and three months after rituximab discontinuation (B).

**Figure 3:**
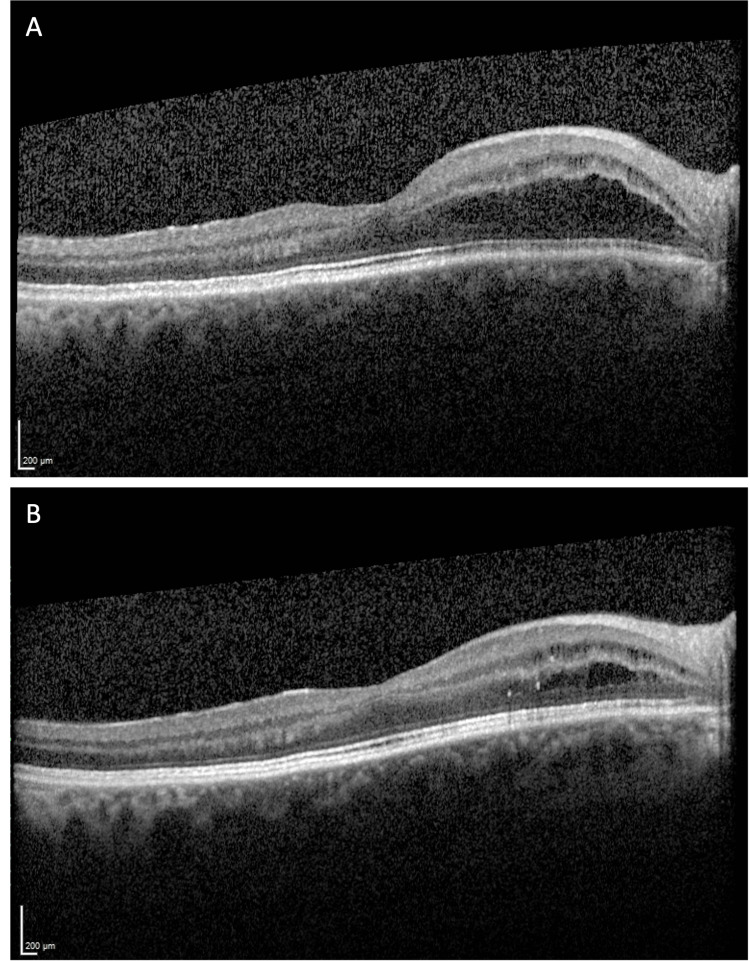
Optical coherence tomography scans of the right eye at the initial presentation (A) and three months after rituximab discontinuation (B).

The first RTX infusion occurred without immediate complications. However, one week later, during the medical assessment before the second infusion, the patient reported bilateral blurred vision and reduced peripheral vision starting a few hours after the first RTX administration. Urgent ophthalmological evaluation confirmed decreased visual acuity (right eye 8/10, left eye 6/10), central neurosensory detachment, and intraretinal and subretinal fluid areas bilaterally, consistent with CME on OCT (Figures 2A-3A).

Etiological studies, including complete blood count, protein electrophoresis, angiotensin-converting enzyme, virus serologies, and immunological studies, ruled out other causes of CME, such as ocular inflammatory diseases, retinal vascular diseases, or retinal vein occlusion. RTX was discontinued (the patient continued only on prednisolone) and lifelong avoidance of RTX was advised. A retinal ophthalmology follow-up was scheduled within three months, during which the patient showed substantial improvement in visual acuity (right eye 10/10, left eye 8/10), along with improvement in OCT; the macular edema was more circumscribed, with only a thin subretinal fluid lamella (Figures 2B-3B). 

Despite receiving only one dose of RTX, the patient reported significant improvement in peripheral edema and showed a loss of five kilograms in the first two weeks post-treatment at an early reassessment. During a follow-up consultation four months after the single RTX dose, while on a corticosteroid taper (0.3 mg/kg/day), the patient remained clinically stable, asymptomatic, denied dyspnea or asthenia and showed no peripheral edema. The laboratory findings demonstrated stabilized renal function, an improvement in serum albumin to a normal range, and a notable reduction in both proteinuria and albuminuria (Table 1).

## Discussion

MGN is the primary cause of nephrotic syndrome in nondiabetic Caucasian adults [1]. It appears in its idiopathic form in approximately 80% of cases and is mainly associated with PLA2Rab; however, it can also be secondary and linked to infections, autoimmune diseases, or neoplasms [1]. While approximately one-third of patients achieve disease remission spontaneously, approximately 30% progress to chronic renal disease, necessitating the initiation of renal replacement therapy. Therefore, the use of immunomodulatory drugs is crucial in managing this disease [1, 3].

Upon admission to the nephrology clinic, our patient had overt proteinuria (>3.5 g/24 h) while on a high-dose ACE inhibitor for more than six months, alongside PLA2Rab > 50 U/ml. Consequently, he presented a high risk of progressing to ESRD, warranting immunosuppression, in line with Kidney Disease Improving Global Outcomes (KDIGO) guidelines [3].

RTX is a monoclonal antibody that directly targets the CD20 antigen on B cells, depleting these cells in peripheral blood [4]; this antibody is one of the recommended drugs for treating MGN [1,3]. Its most frequent adverse effects are infusion-related and fever, pneumonia, febrile neutropenia, and anemia, with ocular reactions reported in less than 1% of the population [5].

CME occurs when excess fluid accumulates in the macular retina [6]. It can be caused by viruses (such as cytomegalovirus associated with AIDS), postocular surgery, retinal vein occlusion, retinal vascular diseases (as in cases of diabetes mellitus and uncontrolled hypertension), hereditary, idiopathic; it can also be drug-related [6]. There was no evidence of the above-mentioned clinical situations in our patient, and there are no reported cases of macular edema related to MGN. The temporal relationship between RTX infusion and symptom onset strongly suggested a pharmacological reaction.

CME is typically managed through the administration of topical non-steroidal anti-inflammatory drugs or intravenous/intravitreal corticosteroid therapy [6]. Previously, five cases of CME have been reported: two patients with Wegener's granulomatosis, two with IgG-4 disease, and one with antibody-mediated rejection of a renal transplant [7-10]. Visual symptoms were observed within a range of hours to one month following the initial infusion of RTX, regardless of the dosage, in the reported cases. In two of these patients, a recurrence of CME was observed after a new RTX infusion, reinforcing the relationship between these two events [7,10]. In all of the previously reported cases, patients received targeted treatment for CME, which resulted in significant improvement-either with triamcinolone injections (subtenon [7] or intravitreal [8-9]), intravenous methylprednisolone [7], or intravitreal dexamethasone injections [10].

This case is the only one in which the patient achieved improvement in visual acuity and almost complete resolution of CME lesions without targeted treatment, solely upon discontinuation of RTX and reevaluation in the short term. Nevertheless, the patient had already been administered oral prednisolone for MGN, potentially influencing the recovery, despite it not being a standard treatment for this pathology. Furthermore, it is noteworthy that this reported case exhibited the least severe visual acuity loss.

Despite the inherent risks of immunosuppressive therapy, RTX has been shown to have consistent effects on regressing primary MGN, reducing proteinuria by approximately 80% in most patients [3]. This is supported by the evolution of MGN in our patient, with a drastic reduction in proteinuria and albuminuria at the follow-up appointment.

## Conclusions

Although exceptionally rare, there have been a few documented cases of cystoid macular edema following rituximab administration. This case stands out due to the recovery of visual acuity and ocular anatomy without targeted treatment, which is unprecedented in the literature.

Therefore, as with any medication, especially immunosuppressants, careful consideration of risk versus benefit and monitoring of treatment-related side effects by clinicians are fundamental.
